# The Efficacy of Transversus Abdominis Plane Blocks in Roux-en-Y Gastric Bypass – a Systematic Review and Meta-Analysis of Randomised Control Trials

**DOI:** 10.1007/s11695-026-08711-4

**Published:** 2026-05-06

**Authors:** Caroline Drumm, Matthew G. Davey, Shane Moore, Taya Keating, Noel E. Donlon

**Affiliations:** 1 Royal College of Surgeons in Ireland, Dublin, Ireland; 2 Connolly Hospital Blanchardstown, Dublin, Ireland; 3 College of Anaesthetists of Ireland, Dublin, Ireland; 4 St. James’s Hospital, Dublin, Ireland

**Keywords:** Bariatric surgery, Gastric bypass, Roux-en-Y, Transversus abdominis plane block, TAP block

## Abstract

**Background:**

Transversus abdominis plane (TAP) block is commonly used as an element of multimodal analgesia following abdominal surgery; however, its efficacy in Roux-en-Y gastric bypass (RYGB) remains uncertain.

**Aim:**

To perform a systematic review and meta-analysis of randomised clinical trials (RCTs) to evaluate the effectiveness of TAP block following RYGB.

**Methods:**

A comprehensive search was performed as per PRISMA guidelines. RCTs comparing TAP block with control in adult patients undergoing RYGB were included. Primary outcomes were postoperative pain scores (visual analogues scores (VAS)/numeric rating scale (NRS)) in recovery and at 6, 12, and 24-hours. Data analytics were performed using RevMan v.5.3.

**Results:**

Five RCTs comprising 481 patients were included. TAP block did not significantly reduce pain scores in recovery, at 6-hours, or at 12-hours postoperatively. A significant reduction in pain was observed at 24-hours (mean difference (MD) -0.57, 95% CI -0.96- -0.17, *p* = 0.005, I^2^ = 92%). Patients receiving TAP block were significantly less likely to require breakthrough opioid analgesia (odds ratio (OR) 0.09, 95% confidence interval (CI) 0.02–0.32, *p* = 0.0003, I^2^ = 51%). TAP block was also associated with earlier ambulation (MD -1.65, 95% CI -2.32- -0.98, *p* < 0.00001, I^2^ = 0%). No significant differences were observed in PONV or LOS.

**Conclusion:**

While TAP block provided limited benefit in early postoperative pain control in patients undergoing RYGB, it was associated with reduced ‘breakthrough’ opioid requirements, earlier mobilisation, and a significant analgesic effect at 24 h. These findings support the use of TAP blocks as an opioid-sparing adjunct within enhanced recovery after bariatric surgery pathways.

**Supplementary Information:**

The online version contains supplementary material available at 10.1007/s11695-026-08711-4.

## Introduction

Bariatric surgery represents the most effective treatment for sustained weight loss and subsequent improvement or complete resolution of these associated metabolic conditions [1, 2]. Roux-en-Y gastric bypass (RYGB) is a commonly performed bariatric operation worldwide and has demonstrated significant long-term benefits in management of obesity and metabolic syndrome [3–6].

Despite advances in minimally invasive surgery, post-operative pain remains an issue following laparoscopic RYGB. Effective pain control in this cohort is particularly important, as inadequate analgesia may impair respiratory function, delay mobilisation and prolong recovery [7]. Furthermore, those with obstructive sleep apnoea have an increased risk of opioid-related adverse events, such as respiratory depression [8]. As a result, strategies that minimise opioid use while maintaining adequate analgesia are a key focus of perioperative care in bariatric surgery.

Enhanced Recovery After Bariatric Surgery (ERABS) protocols advocate a multimodal approach to analgesia in order to optimise postoperative recovery while reducing opioid requirements [9, 10]. Regional anaesthetic techniques have therefore gained popularity as adjuncts to systemic analgesia. The transversus abdominis plane (TAP) block is a regional anaesthetic technique that involves injection of local anaesthetic into the fascial plane between the internal oblique and transversus abdominis muscles, thereby blocking the sensory nerves supplying the anterolateral abdominal wall [11]. TAP blocks can be performed under ultrasound or laparoscopic guidance and have been shown to provide effective analgesia in a variety of abdominal procedures, including appendicectomy, cholecystectomy and sleeve gastrectomy [12–15].

However, the role of TAP block in RYGB remains uncertain. As outlined in previous reviews, TAP blocks and other regional techniques have been shown to be effective in reducing pain in the post-operative setting following sleeve gastrectomy alone and in all laparoscopic bariatric surgeries [12, 16, 17]. To our knowledge, there is no previous meta-analysis assessing efficacy TAP blocks in RYGB alone.

Given the increasing emphasis on opioid-sparing analgesia within enhanced recovery pathways, clarification of the potential benefits of TAP block is vital. As such, the aim of this study was to perform a systematic review and meta-analysis of randomised control trials (RCTs) to evaluate the effectiveness of TAP block in patients undergoing RYGB surgery, with particular focus on postoperative pain scores and opioid requirements.

## Methods

This systematic review was conducted in accordance with the Preferred Reporting Items for Systematic Reviews and Meta-Analyses (PRISMA) 2020 guidelines [18]. This study was prospectively registered with the International Prospective Register of Systematic Reviews (PROSPERO-CRD42025638050).

### Population, Intervention, Comparison and Outcome Framework

The review question was structured using the Population, Intervention, Comparison and Outcome (PICO) Framework [19]:

#### Population

Adult patients (≥ 18 years) indicated to undergo RYGB surgery for any indication.

#### Intervention

Any patients randomised to receive TAP block.

#### Comparison

Any patients randomised to receive control.

#### Outcomes

- The primary outcomes of interest include visual analogue score/numerical rating scale (VAS/NRS) measurements performed in recovery, 6-hours, 12-hours and 24-hours post-operatively.
- The secondary outcomes of interest are the number of patients requiring opioid analgesia, post-operative nausea and vomiting (PONV), time to ambulation (measured in hours), length of hospital stay (LOS, measured in hours).

### Search Strategy

A comprehensive search of literature published up to November 2025 was performed using the following databases: PubMed, Embase and Cochrane Library. The search terms included (transversus abdominis plane block), (Roux-en-Y), (gastric bypass) and (bariatric surgery). The search strategy combined MeSH and the above free-text terms linked by Boolean operators. Included studies were limited to the English language and were not restricted by year of publication. The final search was conducted in November 2025. All duplicate studies were removed before titles were screened. Two collaborators examined potentially eligible studies, initially conducting a title and abstract review followed by a full-text review to determine eligibility for inclusion. Reference lists were cross-referenced to ensure that no studies were overlooked. Any discrepancies were resolved through joint agreement between both collaborators.

### Inclusion Criteria

The included trials were prospective RCTs that investigated TAP block versus control in gastric bypass surgery. The RCTs must have included at least one of the following outcomes: VAS/NRS measurements performed in recovery, 6-hours, 12-hours and 24-hours post-operatively, post-operative analgesic requirements and/or the number of patients requiring opioid analgesia. All non-randomised trials were excluded.

### Extraction of Data

Characteristics of the baseline study were extracted, including first author name, year of publication, study design, sample size, patient demographics, details of interventions and controls, post-operative analgesic regimens and outcomes. Data were extracted independently using a standardized data extraction form. Disagreements were resolved by discussion, with resolution by a third reviewer, as required.

### Statistical Analysis

Descriptive statistics were used to determine associations between TAP block and control and the primary and secondary outcomes of interest, as appropriate. This involved the use of the Fisher’s Exact (†) and independent samples t-tests (‡), where appropriate [20]. Outcomes measured using VAS and NRS were combined given their similarities to facilitate meta-analysis. All tests of significance were two tailed with *P* < 0.05 representing statistical significance. Descriptive statistics were performed using the Statistical Package for Social Sciences (SPSS) version 26 (International Business Machines Corporation, Armonk, New York). Thereafter, relevant outcomes for patients randomised to TAP or control were expressed as dichotomous, reported as odds ratios (ORs) with their corresponding 95% Confidence Intervals (CIs), following estimation using the Mantel-Haenszel method. Continuous data was analysed using inverse variance methodology, with mean differences (MD) and CIs reported. Given the heterogeneity via including trials from surgical research institutions across the world, random effects models were applied irrespective of reported heterogeneity (I²). Meta-analyses were performed using Review Manager (RevMan), Version 5.4 (Nordic Cochrane Centre, Copenhagen, Denmark).

### Quality Assessment

Risk of bias was assessed independently by two reviewers using the Cochrane Risk of Bias 2 (ROB2) tool across the domains of randomisation process, deviations from intended interventions, missing outcome data, outcome measurement, and selective reporting [21].

## Results

**Fig. 1:**
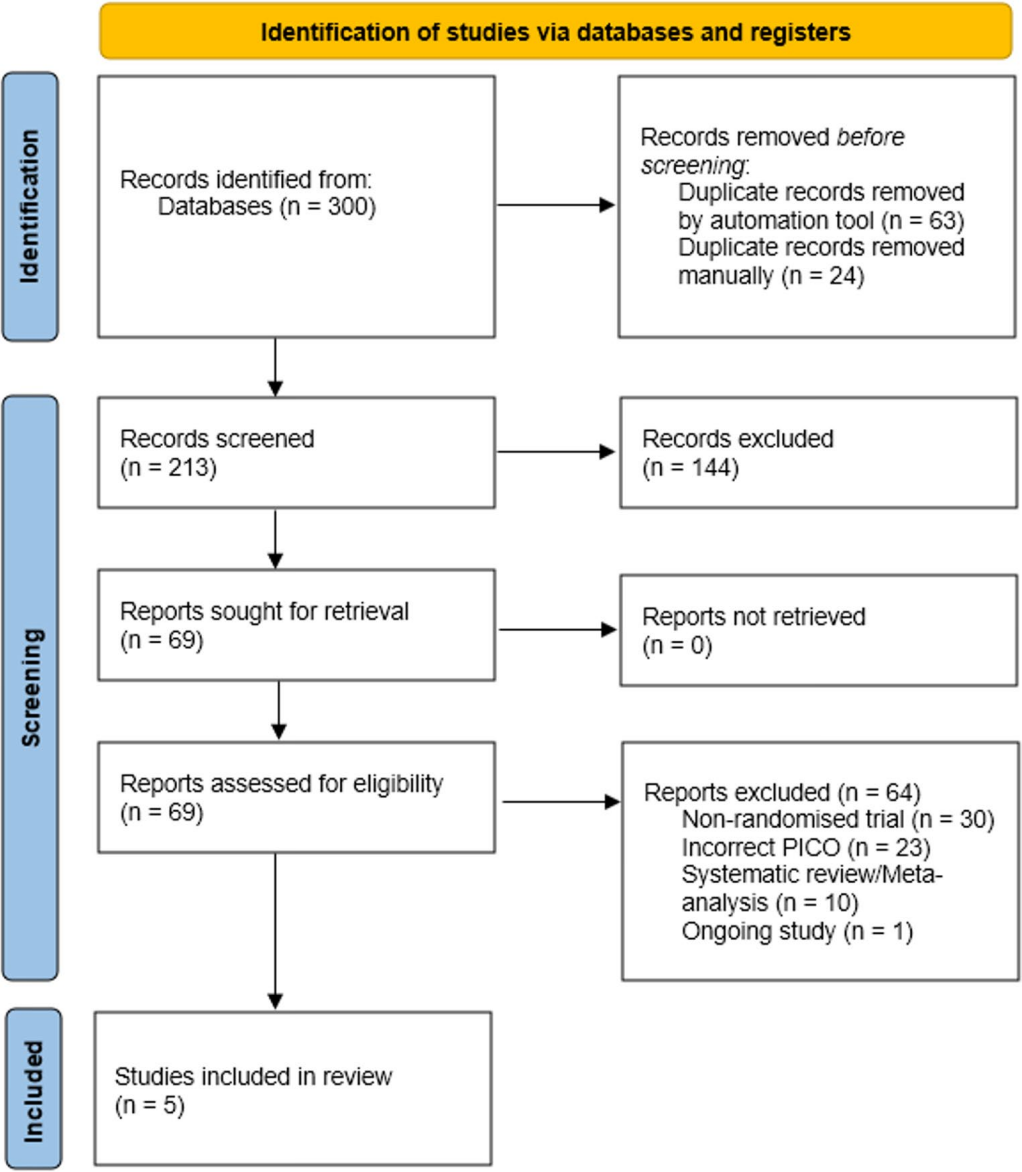
PRISMA Flow Diagram

A total of 300 studies were initially identified following the database searches. Of these, 213 study titles were screened, following exclusion of 87 duplicate articles. Following completion of full text screening of 69 studies, 5 studies were found to meet the aforementioned eligibility criteria (Fig. 1). All included studies were RCTs published between 2013 and 2025. All RCTs reported on outcomes in relation to patients undergoing RYGB only. The study characteristics of these RCTs are outlined in Table 1.

**Table 1:** Study Characteristics

	Intervention	Control	Local Anaesthetic	Post-op Analgesia	Primary Outcomes	Inclusion Criteria
Sinha (2013) [22]	US-guided TAP block	US-guided TAP with saline	40mls of 0.375% ropivacaine	Tramazac hydrochloride	Requirement of Tramazac hydrochloride in first 24 h	Age over 18, BMI > 35 kg/m^2^
Albrecht (2013) [23]	US-guided TAP block + PSI	PSI with LA	TAP − 60mls of 0.25% bupivacaine with adrenaline PSI − 20mls 0.25% bupivacaine with adrenaline	Paracetamol 1 g QDS PO, oxycodone 5–10 mg PO 4hrly and in cases of severe pain morphine 2–6 mg IV 3hrly	Cumulative opioid consumption during first 24 h	ASA I-III, age between 18–70
Ruiz-Tovar (2018) [27]	Laparoscopic-guided TAP block	PSI with LA	TAP – 30mls 0.25% bupivacaine PSI – 30mls 0.25% bupivacaine	IV metamizole 2 g/8hrly and paracetamol 2 g/8hrly, alternating every 4 h 5 mg of SC morphine given with VAS > 5	Pain scores, morphine requirements in first 24 h	BMI > 35 kg/m^2^ and at least one obesity related condition or a BMI > 40 kg/m^2^
Gogokhia (2024) [28]	Opioid-free anaesthesia + TAP block	GA with opioids		2 ml Dexalgin Inject 25 mg/ml +/- 1 ml Promedol 20 mg/ml	Post-operative analgesia requirements	
Cataldo (2025) [26]	Laparoscopic-guided TAP + PSI	Laparoscopic-guided TAP with saline + PSI with LA	TAP – 30mls of 0.5% ropivacaine PSI – 10mls of 0.5% ropivacaine	Paracetamol 1 g IV QDS Ketorolac 30 mg IV (max 90 mg) if NRS > 6 and if severe pain, morphine 2 mg IV (max 10 mg)	Pain scores	Age 18–65, BMI > 35 kg/m^2^ and at least one obesity related condition or a BMI > 40 kg/m^2^

*US* ultrasound, *LA* local anaesthetic, *PSI* post-site infiltration, *GA* general anaesthesia

### Quality Assessment

Two studies were found to have low risk of bias, with two others showing some concerns for bias. One paper was deemed to have a high risk of bias. The Risk of Bias 2 (ROB2) assessments are outlined in Table 2.

**Table 2:** Risk of Bias 2 Assessment

	RSG	AC	BOP	BOOA	IOD	SR	OB	Overall Risk
Sinha (2013) [22]	Low	Low	Low	Low	Low	Unclear	Low	Low
Albrecht (2013) [23]	Low	Low	Low	Low	Low	Unclear	Low	**Low**
Ruiz-Tovar (2018) [27]	Unclear	Unclear	High	High	Low	Unclear	Unclear	**Some concerns**
Gogokhia (2024) [28]	Unclear	Unclear	Unclear	Unclear	High	High	Unclear	**High**
Cataldo 2025 [26]	Low	Low	Low	Low	Unclear	Unclear	Low	**Some concerns**

*RSG* random sequence generation, *AC* allocation concealment, *BOP* blinding of participants, *BOOA* blinding of outcome assessment, *IOD* incomplete outcome data, *SR* selective reporting, *OB* other bias

### Demographics

This analysis included 481 patients, of whom 241 (50.1%) were randomised to receive TAP block. Patient demographics are presented in Table 3.

**Table 3:** Patient Demographics

		Sample size	Gender (M: F)	Age at surgery (years)	BMI (kg/m2)
Sinha (2013) [22]	TAP	50		39.9	48.1
	Control	50		39.1	45.6
Albrecht (2013) [23]	TAP	27	7:20	44.8	49.3
	Control	30	4:26	38.8	48.9
Ruiz-Tovar (2018) [27]	TAP	70	30:40	41.9	47.4
	Control	70	30:40	41.7	46.5
Gogokhia (2024) [28]	TAP	53	22:81	40.9	48.4
	Control	50			
Cataldo (2025) [26]	TAP	42	12:30	43.1	41.8
	Control	42	13:29	45.5	42.1

### Post-Operative Pain Scores

**Fig. 2:**
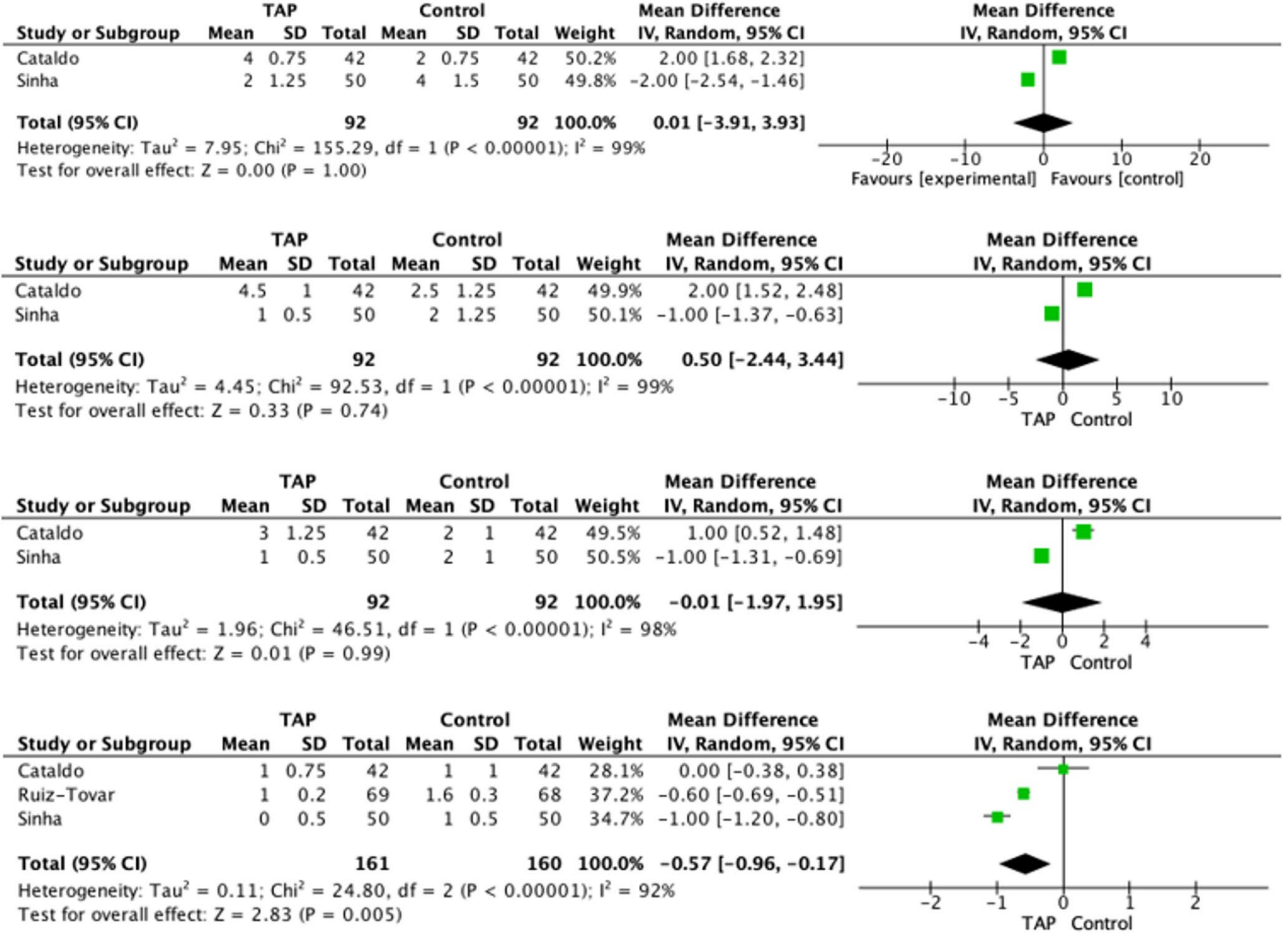
Pain Scores (**A**) in recovery, (**B**) at 6-hours, (**C**) at 12-hours and (**D**) at 24-hours

Two of the five studies (*n* = 184) reported on pain scores in the immediate post-operative recovery period, at 6-hours post-operatively and at 12-hours post-operatively. There was no significant difference between the TAP and control groups in recovery (MD 0.01, 95% CI −3.91-3.93, *p* = 1.00, I^2^ = 99%), at 6-hours (MD 0.50, 95% CI −2.44-3.44, *p* = 0.74, I^2^ = 99%) or at 12-hours (MD −0.01, 95% CI −1.97-1.95, *p* = 0.99, I^2^ = 98%) (Fig. 2).

Three studies (*n* = 321) reported on pain scores at 24-hours. At this time point, there was a significant reduction observed in post-operative pain seen in the TAP group, when compared to the control group (MD −0.57, 95% CI −0.96- −0.17, *p* = 0.005, I^2^ = 92%) (Fig. 2).

### Breakthrough Opioid Use

**Fig. 3:**
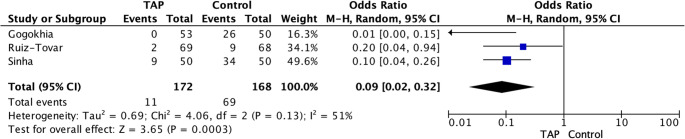
Breakthrough opioid use

Three studies reported on breakthrough opioid use, with a significant difference observed in favour of those in receipt of TAP block (OR 0.09, 95% CI 0.02–0.32, *p* = 0.0003, I^2^ = 51%) (Fig. 3).

### Time to Ambulation

**Fig. 4:**
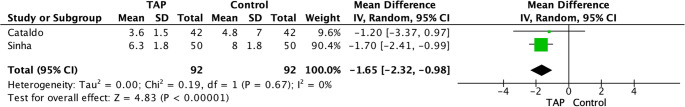
Time to Ambulation

Two of the five studies report on post-operative time to ambulation, with a significantly shorter time period to ambulation seen with the TAP group, in comparison with the control group (MD −1.65, 95% CI −2.32- −0.98, *p* < 0.00001, I^2^ = 0%) (Fig. 4).

### Post-operative Nausea and Vomiting (PONV)

Two studies investigated levels of PONV at 24-hours post-operatively, with no significant difference between TAP and control groups (OR 2.01, 95% CI 0.66–6.12, *p* = 0.22, I^2^ = 0%) (See Supplementary Material).

### Length of Stay (LOS)

With three of the five studies reporting on LOS, there was a no statistically significant difference seen between the groups (MD −7.68, 95% CI −22.02-6.66, *p* = 0.29, I^2^ = 88%) (See Supplementary Material).

## Discussion

This systematic review evaluated five RCTs with a total of 481 patients, 241 of whom received TAP block following their laparoscopic gastric bypass surgery. There was no significant reduction in post-operative pain scores in recovery, at 6-hours, or at 12-hours, however, a significant reduction in pain scores following TAP block was observed at 24-hours. Interestingly, those who received TAP blocks were significantly less likely to require breakthrough analgesics; this was observed in both the number of patients requiring breakthrough analgesia, and in post-operative opioid use in milligrams reported in several studies [22, 23]. These findings suggest that, although pain was still present, their pain control was sufficient to not request or require breakthrough analgesia. While multi-modal analgesia is important, this suggests that TAP blocks are an important opioid-sparing analgesic option in these patients, who are high risk for associated respiratory complications, such as hypoventilation and exacerbation of OSA [8]. As such, the inclusion of TAP block works within the ERABS pathway [9].

There was a significantly shorter time to ambulation seen in the TAP group when compared to the control group. This may contribute to a smoother post-operative course, with improved mobility reducing the risk of post-operative respiratory infections and venous thromboembolism in this high-risk cohort [24]. These findings, again, remain in line with ERABS recommendations, which emphasize early mobilisation as a key component of post-operative recovery.

Bariatric surgery is performed for patients living with obesity and metabolic syndrome, with other co-morbidities, including hypertension, type 2 diabetes mellitus and obstructive sleep apnoea [25]. With this high-risk and comorbid cohort, optimisation of peri-operative care and the reduction of post-operative pain and complications is imperative. Effective post-operative pain control plays a key role in improving patient outcomes and satisfaction. Adequate analgesia helps facilitate adequate respiratory utilization, reducing the risk of atelectasis and respiratory complications, while also enabling early mobilisation which is important in the prevention of thrombosis-related events [7]. Importantly, the results of this study indicated that TAP block facilitated earlier mobilisation in the post-operative phase, which in essence, may translate to a reduction in the potential negative sequalae of immobility in the early phases of patient recovery.

Multimodal analgesia makes up a central component of the ERABS protocol [9]. TAP blocks involve the injection of local anaesthetic into the facial plane between internal oblique and transversus abdominus muscles of the abdominal wall usually under laparoscopic or ultrasound guidance. Ultimately, this technique aims to block pain sensation of the anterior rami of lower thoracic and upper lumbar nerves supplying the anterior abdominal wall, depending on the location of injection [11]. Within the included studies, two trials used ultrasound-guided TAP block, while others used laparoscopic-guided techniques [22, 23, 26, 27]. Additionally, Cataldo et al. combined TAP block with port-site infiltration, and Gogokhia et al. evaluated TAP block within an opioid-free anaesthesia framework, further contributing to heterogeneity in study design, which is of course important to note [26, 28].

As previously detailed, no significant reduction in pain scores was observed in the early post-operative phase, but a significant reduction was seen at 24-hours. These findings may be explained by the pharmacokinetic profile of bupivacaine and ropivacaine, whose half-lives are approximately 3–6 h [29]. Additionally, laparoscopic gastric bypass surgery, in particular, is associated with significant post-operative pain, thought secondary to significant visceral pain associated with manipulation of the stomach and small bowel [30]. This may explain the limited benefit seen in post-operative pain scores, as laparoscopic gastric bypass involves small abdominal wall incisions but significant visceral manipulation, which may limit the contribution of abdominal wall blockade in the early post-operative period.

It is also important to note the varying controls used, with three of the five papers comparing port-site infiltration to TAP block with or without concurrent port-site infiltration [23, 26, 27]. Introducing a second active abdominal wall anaesthetic agent may have attenuated differences seen between the groups. Sinha et al. used saline-only TAP block as a placebo in their study, maximising the observable effect size of TAP block by minimizing competing regional analgesia [22]. Additionally, variability was seen within baseline post-operative analgesia regimens, which may have contributed to the absence of consistent early post-operative benefit between studies.

There were no differences seen in PONV and LOS. This is likely due to the multifactorial nature of these outcomes. PONV, while can be secondary to pain, could also be affected by anaesthesia, opioid administration and patient susceptibility [31]. Again, in terms of LOS, a generally short admission period was seen across the studies, making it difficult to delineate a significant difference secondary to TAP blocks alone.

This main strength of this review is the sole inclusion of RCTs within a specific surgical population, enhancing validity and applicability to our specific cohort. However, there are a number of limitations that should be acknowledged. Firstly, the total number of included trials was small, with relatively modest sample sizes across studies. Secondly, there was considerable heterogeneity between trials, including differences in pain scoring systems, control interventions, techniques used to perform TAP block, and postoperative analgesic regimens. Finally, one of the included studies was found to be high risk of bias and was published only in abstract form. This limits the ability to fully assess the quality of the study, while also increasing the potential risk of selective reporting bias.

In conclusion, while TAP block provided limited benefit in early post-operative pain control in patients undergoing RYGB, it was associated with reduced breakthrough opioid requirements, earlier mobilisation and a significant analgesic effect at 24 h. These findings support the use of TAP block as an opioid-sparing adjunct within ERABS pathways. Given the substantial heterogeneity and limited number of trials, further high-quality multi-centre RCTs may be required to fully ascertain the true efficacy of TAP block in patients undergoing RYGB.

## Supplementary Information

Below is the link to the electronic supplementary material.

Supplementary Material 1 (DOCX 162 KB)

## Data Availability

No datasets were generated or analysed during the current study.
