# Supplementary figures and images for: The Efficacy of Transversus Abdominis Plane Blocks in Roux-en-Y Gastric Bypass – a Systematic Review and Meta-Analysis of Randomised Control Trials
Source: Obes Surg. 2026 May 6;36(6):3350–8. doi: 10.1007/s11695-026-08711-4 (PMC13249660; doi:10.1007/s11695-026-08711-4)

**Supplementary Material -** PONV and LOS:


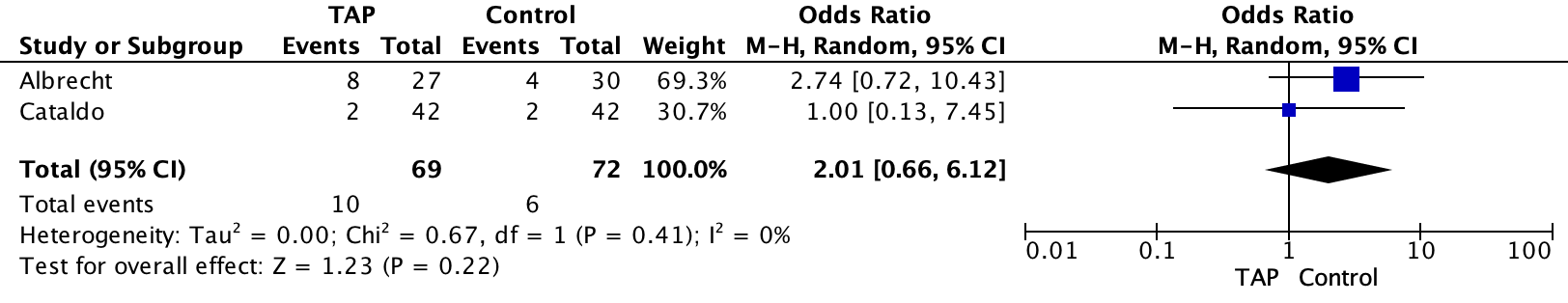


Figure 5. PONV


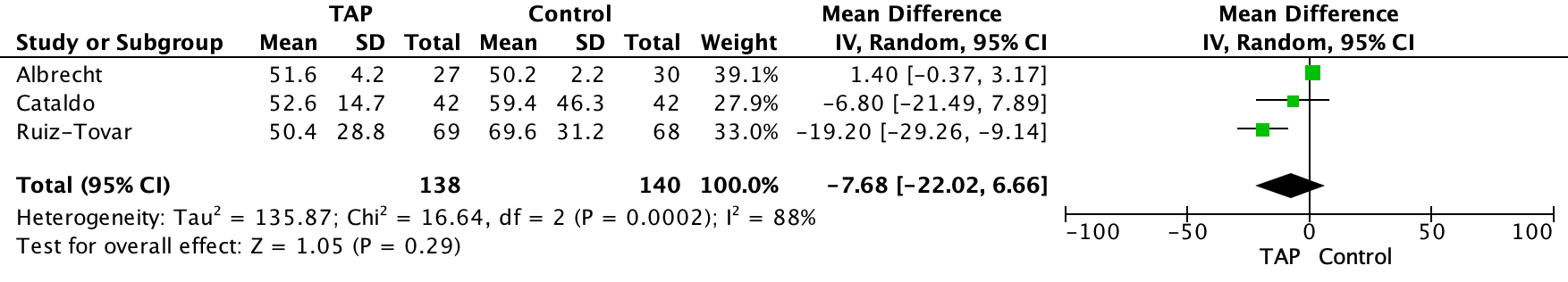


Figure 6. Length of Stay

Supplement: Supplementary file 1 — Supplementary Material 1 (DOCX 162 KB) [file 11695_2026_8711_MOESM1_ESM.docx]
